# Iron Profile and Inflammatory Status of Overweight and Obese Women in Sari, North of Iran

**Published:** 2017-04-01

**Authors:** Ramin Shekarriz, Mohammad Mehdi Vaziri

**Affiliations:** 1 Gastrointestinal Cancer Research Center, Mazandaran University of Medical Sciences, Sari, Iran; 2 Department of Internal Medicine, Mazandaran University of Medical Sciences, Sari, Iran

**Keywords:** Iron deficiency, Obesity, Iron profile, Inflammatory status

## Abstract

**Background** **: **It has been suggested that inflammatory state due to obesity can lead to alteration in iron metabolism. Women in reproductive age are at higher risk of iron deficiency. In this study, we aimed to evaluate inflammatory status and iron markers in young overweight and obese women.

**Subjects and Methods** **:** In this study, 120 young and healthy women with a BMI ≥ 25 kg/m2 were enrolled. Biochemical data including iron profile and inflammatory markers were analyzed using mean ± standard deviation or median (interquartile range) and multivariate multiple regression model via MANOVA.

**Results** **: **Iron deficiency anemia (hemoglobin < 120 g/l) and iron deficiency without anemia (serum ferritin<30.0 mg/l) were detected in 21.67% and 33.33% of participants, respectively. Multivariate modeling showed that BMI was a significant predictor of transferrin saturation (p = 0.037), CRP (p = 0.013), soluble transferrin receptor (p=0.045), and soluble transferrin receptor/ ferritin ratio (0.015).

**Conclusion:** The results of this study supported the positive association between obesity and inflammation and mild changes in iron markers.

## Introduction

 Obesity and high body fat content are as major risk factors for many chronic diseases. Obesity increases the risk of type II diabetes, hypertension, cardiovascular disease, stroke, dyslipidemia, osteoarthritis, gynecological problems, sleep apnea and respiratory disorders.^1^^-^^3^ Therefore, an increase in prevalence of obesity leads to increased health-care costs for prevention and treatment of obesity-related problems.^3^ Obesity and iron deficiency anemia are common diseases worldwide and despite global development in nutrition science and economy status, they still involve billions of people in the world.^2^ Adipose tissue contributes as an endocrine organ that can participate in inflammatory processes by secreting pro-inflammatory cytokines, named adipokine. Therefore, obesity can be considered as pre-inflammatory condition characterized by systemic mild chronic inflammation. This inflammatory status may play an important role in the pathogenesis of obesity-related disorders, especially iron deficiency.^4^First reports on the correlation between iron status and obesity were published more than 40 years ago**. **These studies observed lower serum iron concentration in obese adolescents compared to peers with normal weight.^5^ During the past decades, several studies revealed that inflammation caused by obesity, similar to anemia of inflammation can lead to defects in ironhandling.^2^^,^^6^So far, few studies have investigated the body weight as a relevant factor to iron status.^7^^,^^8^ A pioneer clinical study that evaluated obesity and iron deficiency was done by Bekri et al.^9^ For the first time, they showed that hepcidin was expressed not only in the liver but also within adipose tissue at both messenger RNA (mRNA) and protein levels. Hepcidin mRNA was higher in all obese patients selected for bariatric surgery.^9^

Generally, pre-menopausal women are at higher risk for obesity. Regular menstrual iron loss, low iron intake and restrictive diets for losing weight increases the risk of iron deficiency among this group. Therefore, it is understood that a combination of inflammatory factors, diet and age can disturb iron status in overweight women.^2^ However, we must consider that obesity even without anemia has an adverse impact on physical function, mental health and cognitive performance. On the other hand, maternal iron deficiency anemia (IDA) has adverse effects on birth consequences, neonatal cognitive development and increased risk of postpartum depression.^2^ Therefore, insufficient iron level is an important issue which necessitates further studies of iron status among this population. At present, few surveys have investigated iron deficiency caused by obesity among pre-menopausal women. In this study, we aimed to evaluate iron status and its association with obesity by using a wide spectrum of iron biomarkers in a group of young overweight or obese women. In contrast to previous studies which mostly focused on children, adolescents or the elderly, we examined correlation between obesity-related inflammation and iron status independent of any other disease among young women. 

## SUBJECTS AND METHODS

 We planned a cross-sectional study to investigate iron profile and inflammatory status. Our study population included 120 overweight or obese women (18-45 years) with a measured body mass index (BMI) ≥ 25 Kg/m² who referred to private or governmental Nutrition Clinics for losing weight in Sari, Iran, 2014 to 2015. The local clinical Research Ethics Committee approved the study protocol and all cases confirmed written informed consent for the use of their clinical data just for research purposes before participation. Exclusion criteria were the presence of diabetes, hypothyroidism, cushing, polycystic ovarian syndrome (PCOS), hepatic disease including hemochromatosis, renal, autoimmune or metabolic disorders, malignancy, pregnancy or breast feeding, any infectious or inflammatory disease, hormonal contraceptive methods, or intake of specific drugs or substances which influence iron profile, body weight and inflammatory conditions such as vegetarianism, zinc supplements, cigarette smoking and bariatric surgery. Those who reported iron supplementation, blood transfusion or donation recruited in the study after a washout period of 3 months. Subjects that received vitamin supplements, fish oil and minerals (except iron or zinc) were included in the study after a washout period of 2 weeks. To rule out diabetes and hypothyroidism, we used screening tests of FBS (Fasting Blood Sugar) and TSH (Thyroid Stimulating Hormone). Clinical exam was used to rule out cushing disease and PCOS. For other items of exclusion criteria, participants’ self-reports were confined. In the clinic, heights were examined by metal meter (SOEHNLE, Kish Island, Iran), weight by scale (Beurer GmbH, Ulm, Germany), and waist circumference by cloth scaled meter. BMI was calculated by dividing body weight (Kg) on the square of height (meter). The vein blood samples were collected and total iron binding capacity (TIBC), ferritin, serum iron and Hepcidin, soluble transferrin receptor (sTfR), sTfR/ ferritin index (sTfR-F) as well as FBS, TSH, C-reactive protein (CRP) and hemoglobin (Hb) were measured.

The quantitative variables were expressed by mean ± standard deviation (SD). Pearson correlation coefficient test and multivariate multiple regression model via MANOVA test were performed for correlation analysis of biochemical variables and BMI. *Kolmogorov-Smirnov test *was used to check normality of regression model of variables. Biomarkers were compared among groups of BMI by ANOVA test. Statistical significance was assigned to P values < 0.05. 

## Results

 In this study, 120 obese and overweight women with average age of 32 ± 7 years and BMI 31.08 ± 5.13 (kg/m²) were referred for interdisciplinary evaluation. Iron profile and inflammatory condition of cases are illustrated in Table 1.

**Table 1:** Iron profile and inflammatory condition of overweight and obese women

Biochemical marker	Normal range	Mean ± SD	Prevalence (%)
Hb (gr/l)	120-165	127.20 ± 12.57	
Hb< 120			21.67
Serum iron (µg/dl)	50-170	75.38 ± 32.70	
Serum iron < 50			24.2
Transferin saturation	12-45	18.53 ± 6.21	
Transferin saturation < 12%			20
Serum ferritin (µg/l)	30-165	36.60 ± 43.71	
Serum ferritin < 30			40
sTfR (mg/l)	0.74-2.39	0.71 ± 0.27	
sTfR> 2.39			15
sTfR-F index	0.16-1.8	0.37 ± 0.18	
sTfR-F = 1.8-2.2			9.2
sTfR-F > 2.2			0.8
sTfR-F <1 + Hb< 120 (gr/l)			10
Hepcidin (ng/ml)	0.1-400	1.12 ± 1.04	
Hepcidin> 32.40			0
CRP (mg/l)	< 1	2.76 ± 3.03	
CRP > 10			5.8
TIBC (µg/dl)	330-360	376.18 ± 44.19	
TIBC > 330			36.7

In this study, we found 21.67 % (n=26) of cases with anemia (Hb< 120 gr/L) and 10% (n=12) of cases with AI (Hb< 120 gr/L, sTfR-F < 1). 9.16 % (n=11) of cases were reported as IDA by having both anemia and low ferritin and/or low transferrin saturation levels. Six cases with anemia had both serum ferritin < 30 µg/L and transferrin saturation < 12%. Iron deficiency without anemia (ferritin < 30 µg/L) was found among 33.33% (n=40) of the participants. 15% (n=18) of cases were diagnosed with iron deficiency (sTfR> 2.39 mg/l). The sTfR-F index was considered in three ranges including 1.8-2.2 as iron depletion, > 2.2 as iron deficiency erythropoiesis, < 1 along with Hb< 120 gr/L as anemia of inflammation(AI). Out of the 12 patients with AI, 3 patients had serum ferritin < 30 µg/L and 9 of whom had serum ferritin > 30 µg/L. By measuring ferritin, 48 cases were considered as iron deficiency (ferritin < 30 µg/L), of whom 8 had iron deficiency anemia and 40 had iron deficiency without anemia. Other markers used to evaluate iron profile in obese cases were TIBC and serum iron concentration. Participants with TIBC > 360 μg/dl (63.3%) and serum iron < 50 μg/dl (24.2%) were considered as iron deficiency.

In the present study, the mean concentration of hepcidin was 1.121 ± 1.047 ng/ml and none of the study population had Hepcidin> 32.4 ng/ml. Cases with iron deficiency (n=48) (according to serum ferritin) had Hepcidin< 4 ng/ml. In this study, the mean CRP value was 2.76 ± 3.03 mg/l which was in normal range. Increased CRP (> 10 mg/l) was found in just 5.83% (n=7) of the subjects. In regression model, BMI was a significant prognostic factor for iron markers including transferrin saturation, sTfR-F, sTfR and also inflammatory biomarkers such as hepcidin and CRP (Table 2).

Additionally, in order to evaluate biochemical differences in iron profile according to BMI, cases were stratified into four categories (Table 3). There was a significant correlation between BMI subgroups and serum iron (P = 0.008), transferrin saturation (P = 0.000) and sTfR (P = 0.038) (Table 3).

**Table 2:** MANOVA test results to evaluate correlation between BMI and biochemical variables

Prognostic variable	Wilks Lambda p- value	p-value								
		Hb	Serum iron	Transferin saturation	Serum ferrritin	sTfR	sTfR-F	hepcidin	CRP	TIBC
BMI	0.000	0.006	0.224	0.037	0.124	0.045	0.015	0.000	0.013	0.059
Adjusted R squared		0.819	0.383	0.682	0.514	0.659	0.759	0.938	0.768	0.628

**Table 3:** Comparison between BMI subgroups and Biochemical markers in women with overweight and obesity

	BMI groups (kg/m²)				p-value
	1 (n=29)	2 (n=34)	3 (n=35)	4 (n=22)	
	25-27.49	27.5-29.9	30-34.9	>35	
Biochemical markers (average)					
Hb (gr/l)	124.87	128.3	128.8	126 .01	NS
Serum iron (µg/dl)	84.04	83.07	67.4	67	0.008
Transferin saturation (%)	14.67	16.57	16.44	19.23	0.015
Serum ferritin (µg/l)	56.6	44.42	31.7	29.65	NS
sTfR	0.6	0.58	0.54	0.84	0.038
sTfR-F	0.36	0.31	0.38	0.51	NS
Hepcidin (ng/ml)	0.72	0.49	0.53	0.7	NS
TIBC (µg/dl)	361.17	371.04	383.13	389.95	0.041
CRP (mg/l)	0.8	1.1	2.9	3.65	0.000

NS: Not Significant

## Discussion

 First reports of potential correlation between iron profile and obesity appeared 40 years ago. These studies reported lower levels of serum iron in obese adolescents in comparison to normal weight peers.^5^ During past years, some studies investigated the correlation between iron deficiency and obesity.^10^^, ^^11^ After reports of increasing iron deficiency prevalence among obese and overweight population, new studies have recently started to focus on this issue.^12^Lecube et al.^12^ reported that obese menopause women have higher levels of sTfR compared to non-obese control subjects. They also concluded that BMI is positively and separately correlated with both sTfR-F and sTfR in obese and non-obese women. Menzie et al.^13^ reported a significant difference in lower levels of serum iron and transferrin saturation (serum iron/TIBC) between obese cases and control group. They claimed that fat mass could be a significant negative predictor of serum iron. Yanoffet al.^14^ reported an increase in the prevalence of iron deficiency in obese adults compared to non-obese subjects. They observed a significant lower levels of serum iron and higher levels of sTfR in obese cases. Results of Lecube et al.,^12^ Menzie et al.^13^ and Yanoff et al.’s^14^ studies revealed a significant correlation between serum iron and sTfR with BMI and fat mass in obese adults. In the current study, we found positive significant correlation between BMI with sTfR and sTfR-F. We found that by increasing BMI, serum iron, transferrin saturation and TIBC decreased, but sTfR and CRP increased. Generally, these findings suggest that severe obesity may have a negative effect on iron profile. Moreover, in Yanoff et al.’s^14^ study, the increased ferritin and CRP levels in obese cases revealed that inflammation may play a role in disturbing iron profile. In obese population, ferritin increases in response to inflammation even in iron deficiency states.^14^This indicates the importance of measuring other iron profile biomarkers that are not influenced by acute inflammatory conditions for diagnosis of true iron deficiency (such as sTfR). STfR has higher sensitivity for diagnosis of iron deficiency compared to ferritin in patients who have high ferritin level due to acute phase response.^14^

In this study, we found 48 cases with iron deficiency. Eight of whom had iron deficiency anemia and 40 cases had iron deficiency without anemia. One of the most important markers which has been investigated in many studies is hepcidin.^6^ It is an important regulator of iron homeostasis which inhibits iron absorption in enterocytes and release of recycled iron in macrophages.^6^^,^^15^ This can lead to a drop in iron storage and hypoferremia. Hepcidin synthesis rises during inflammation and high iron level, but decreases in hypoxia and hematopoiesis.^1^

Obesity causes chronic inflammation which associates with expression and release of pro-inflammatory cytokines including interleukin-6 and tumor necrosis factor-alpha (TNF-α).^16^^,^^17^ These pro-inflammatory cytokines may cause release of hepcidin from liver or adipose tissue. Potential role of hepcidin in iron deficiency among obese population was proved by finding high level of hepcidin in cases with severe obesity and positive correlation between adipose tissue expression of hepcidin and BMI.^1^In addition, we demonstrated BMI as an independent prognostic factor for inflammatory markers, CRP and hepcidin, using multivariate multiple regression models. We also proved positive correlation between CRP and BMI, but we did not find correlation between high hepcidin levels and BMI in obese population. 

Cheng et al.^2^showed the presence of inflammation and mild impairment of iron markers with rising in BMI, but they did not find contribution of hepcidin with obesity. Neymotin et al.^3^also showed that, despite a moderate rise in CRP (14%), obesity-associated inflammation has not a clear role in iron deficiency. Also, BMI was not an independent prognostic marker for sTfR and sTfR-F. In this study, BMI was contributed as an important prognostic indicator for serum iron, ferritin, transferrin saturation and CRP. Cheng et al.’s^2^ and Karl et al.’s^18^ findings about existence of a special threshold of obesity for elevation of hepcidin and inflammatory markers are partly consistent with the results of our study.

Bekriet al.^9^ revealed that mRNA expression of human hepcidin gene in adipose tissue significantly correlated with inflammatory markers (IL-6, CRP) and BMI,but expression of liver hepcidin gene did not correlate with CRP. It might be a positive correlation between adipose hepcidin and BMI and a potential role of adipose hepcidin as a possible cause of low iron bioavailability in obese people. Besides this potential role, we should consider that expression of liver hepcidin is 100-folds higher than adipocytes. Also, adipose tissue mass is 20 times more than liver mass.^18^This can be another reason for the hypothesis that there should be a specific threshold of obesity for elevation of hepcidin and iron deficiency.

Tussing-Humphreys et al.’s^19^ study showed that liver hepcidin mRNA expression is about 700 times more than adipose tissue production and strongly correlated with circulatory hepcidin concentrations. In this study, we could not show a positive correlation between CRP and BMI with hepcidin.

Despite what might be expected from adipose hepcidin, we found a positive correlation between hepcidin and iron markers, sTfR and sTfR-F. Increased level of serum ferritin is another reason for elevation of hepcidin. This hypothesis has not been proven yet, but the correlation between hepcidin and ferritin has been showed in many studies.^20^^-^^22^

In this study, 90% of healthy women with high ferritinlevels had lower sTfR. So, we can state that the possible reason for not having higher hepcidin was the absence of any other comorbidities and elevated ferritin in this group. Further studies will be required to confirm the results of the current surveys.

## CONCLUSION

 Our results support positive correlation between obesity and inflammation with mild impairment of some iron markers. Increased BMI is associated with a decrease in serum iron concentration, transferrin saturation, an increase in sTfR and CRP.
